# Small Ruminant Lentiviruses: Genetic Variability, Tropism and Diagnosis

**DOI:** 10.3390/v5041175

**Published:** 2013-04-23

**Authors:** Hugo Ramírez, Ramsés Reina, Beatriz Amorena, Damián de Andrés, Humberto A. Martínez

**Affiliations:** 1 Laboratory of Virology, Genetics and Molecular Biology, FES-Cuautitlán, UNAM C-4 Veterinary, Cuautitlán Izcalli, State of Mexico 54714, Mexico; E-Mail: humberr@unam.mx; 2 Institute of Agrobiotechnology, CSIC-UPNA-Government of Navarra, Ctra. Mutilva Baja *s/n*, Navarra 31192, Spain; E-Mails: ramses.reina@unavarra.es (R.R.); bamorena@unavarra.es (B.A.); ancad@unavarra.es (D.A.)

**Keywords:** SRLV, CAEV, VMV, genetic variability, tropism, diagnosis

## Abstract

Small ruminant lentiviruses (SRLV) cause a multisystemic chronic disease affecting animal production and welfare. SRLV infections are spread across the world with the exception of Iceland. Success in controlling SRLV spread depends largely on the use of appropriate diagnostic tools, but the existence of a high genetic/antigenic variability among these viruses, the fluctuant levels of antibody against them and the low viral loads found in infected individuals hamper the diagnostic efficacy. SRLV have a marked *in vivo* tropism towards the monocyte/macrophage lineage and attempts have been made to identify the genome regions involved in tropism, with two main candidates, the LTR and *env* gene, since LTR contains primer binding sites for viral replication and the *env*-encoded protein (SU ENV), which mediates the binding of the virus to the host’s cell and has hypervariable regions to escape the humoral immune response. Once inside the host cell, innate immunity may interfere with SRLV replication, but the virus develops counteraction mechanisms to escape, multiply and survive, creating a quasi-species and undergoing compartmentalization events. So far, the mechanisms of organ tropism involved in the development of different disease forms (neurological, arthritic, pulmonary and mammary) are unknown, but different alternatives are proposed. This is an overview of the current state of knowledge on SRLV genetic variability and its implications in tropism as well as in the development of alternative diagnostic assays.

## 1. Introduction

Small ruminant lentiviruses (SRLV), which include caprine arthritis encephalitis virus (CAEV) and visna/maedi virus (VMV), also called ovine progressive pneumonia virus (OPPV), cause a progressive multisystemic chronic disease clinically characterized by wasting (visna); breathing difficulty (maedi) associated with pneumonia; encephalitis; arthritis and/or mastitis that affect considerably animal welfare and production. The economic effect of SRLV, often underestimated and still under study, depends on factors related to environment, breed, individual susceptibility, production system, farming practices and age of culling [1,2]. The productive impact is partly due to the premature removal of diseased animals and the consequent increase in the replacement rate [3]. In addition, SRLV infected sheep have shown in some studies decreased fertility and number of lambs per birth, as well as decreased birth weight and decreased weight gain from birth to weaning in progeny of seropositives [4,5]. However, there are studies not claiming any impact of this infection in prolificacy and weight at birth or weaning [6,7,8]. Differences in disease status of the animals and in severity of mammary gland lesions might explain the discrepancies. Animals with advanced disease present a significantly reduced body weight at slaughter, and their carcass may not be qualified for consumption [9]. Furthermore, production losses in adult females may result from decreased milk production accompanied by loss of lambs in the first week of life or a weight shortfall at weaning. SRLV infections represent a serious threat to production in small ruminants especially in intensive milk production systems, because SRLV infection occurs primarily in these systems [2]. The impact of SRLV-induced mastitis on production is also a controversial issue. Some studies do not detect differences in the quantity and quality of milk from infected and uninfected goats [10,11,12]. Other reports show a reduction in milk production (9% in goats of the Murciano-Grenadine breed) without altering milk quality [13]. Finally, there are reports showing a 15% decrease in milk production, associated with low quality by reducing the fat content [14,15]. In dairy sheep, a mean annual decline in milk production and milk fat percentage of 3.2 and 2%, respectively, has been observed [16]. In addition, SRLV infection in the small ruminants may negatively affect the quality of the milk, and it appears to trigger an increased number of somatic cells [15,17].

The intake of infected colostrum and milk by the offspring constitutes a major route of SRLV transmission, but the virus is also transmitted via the respiratory route, especially upon close contact, particularly under intensive housing or grazing conditions [2,18,19,20,21,22]. Both routes of transmission are not mutually exclusive, and the most probable scenario is that, after ingestion of infected colostrum, lambs are further exposed to the virus when raised together with infected adult animals (horizontal transmission). The horizontal transmission route has been widely accepted as responsible for SRLV spread between different geographic regions through programs involving export and exchange of goats and sheep [2]. In fact, the presence of visna/maedi was first described after importing Iceland 20 Karakul sheep from Halle, Germany in 1933 to improve Icelandic local breeds [23], and there are different documented examples that depict SRLV spread through the import of animals from Germany to Greece, Sweden to Finland, Denmark to Norway, Holland to France, Denmark to Scotland, Scotland to Canada, France to England, England to Hungary, Switzerland to USA, and USA to Mexico [2,24,25,26,27]. In the search for SRLV ancestors, recent findings reveal that, together with humans, domestic animals including goats or sheep have travelled from the Fertile Crescent to different countries through the Mediterranean Sea. Viruses infecting those animals could have been the original ancestors of SRLV [28,29,30].

## 2. Viral Genetic Variability Sources

As observed in other lentiviruses, the SRLV proviral genome consists in two identical positive-sense single-stranded RNA subunits (8.4–9.2 kb). Both contain structural (*gag*, *pol* and *env*) and regulatory (*vpr-like*, *vif* and *rev*) genes flanked by non-coding long terminal repeat regions (LTRs) [28]. Despite the high evolution rate of lentiviruses, many elements in the lentiviral genome are conserved over time. One of the most conserved regions of the lentiviral genome is the RNAt^lys^ primer binding site (PBS-GAACAGGGACUUGAA), where the host lysine transfer RNA hybridizes to the viral RNA genome, serving as a primer for reverse transcription. The polypurine tract, Rev responsive element (RRE) and other elements involved in replication and packaging of the viral genome are also conserved at different degrees among lentiviruses [31]. The *gag* and *pol* genes and some regions of the *env* gene are relatively conserved, but others, such as those encoding Env surface protein sites able to bind antibodies, are highly variable. Genetic diversity displayed as viral quasi-species is one of the hallmarks of retroviral infection. The concept of viral quasi-species was first proposed by Manfred Eigen [32] and is defined as a set of viruses found in an infected individual [33]. Under certain circumstances of selective pressure such as that exerted by the immune system, the frequency of genetic forms in the viral population can shift. An “archive” of earlier forms of the virus is retained in proviral DNA and these forms may re-emerge. The extent of genetic diversity within a quasi-species depends on a complex set of factors, including high viral turnover, high mutation rates, retroviral recombination and selection by the host immune system until the limits of genetic and phenotypic constraints to variation [33,34,35,36].

### 2.1. Mutation

Mutations are the substrate for natural selection and underpin the ability of lentiviruses to evade the immune system. Like in other retroviruses, most SRLV mutations are introduced at the reverse transcription stage of the viral life cycle. The most prominent source of variation is attributed to the reverse transcriptase (RT) enzyme itself, which due to the lack of a proofreading capability leads to a high error rate (0.2–2 mutations per genome per cycle) [33,37]. This extremely low fidelity could explain the extremely high levels of genetic variation observed *in vivo*. 

However, the genetic heterogeneity observed *in vivo* cannot be fully attributed to the low fidelity of RT. The minority of subpopulations in the mutant spectrum of the quasi-species viral variants that were dominant *in vivo* early in the evolutionary lineage of a virus can also influence the subsequent evolution of the quasi-species population [35]. In addition, early investigations into the mutation rate of human immunodeficiency virus (HIV) uncovered hypermutated retroviral genomes, where up to 40% of all available guanine bases are substituted by adenines [38]. It is now appreciated that this type of hypermutation is the result of cytosine deamination by members of the APOBEC family of nucleic acid editing enzymes [39,40]. APOBEC proteins are packaged into lentiviral virions and associate with the reverse transcription complex in the target cell, where they deaminate cytosine residues to uracyl in the single-stranded DNA minus strand, leading to G-to-A mutation in the plus strand. The cytosine deamination does not occur randomly, since APOBEC family members have distinct dinucleotide preferences. Furthermore, terminally differentiated cell types, such as macrophages, have imbalanced intracellular dNTP pools, with an excess of dUTP (uracyl) [41]. Uracyl is a natural base in RNA, but is not normally found in DNA. However, it can be incorporated into DNA due to the inability of RT to distinguish between dTTP and dUTP. Consistent with an important role for uracyl in the retroviral life cycle, many macrophage-tropic non-primate lentiviruses (such as SRLV) encode a deoxyuridine 5'-triphosphate nucleotidohydrolyase (dUTPase), which catalyzes the conversion of dUTP to dUMP, maintaining a low dUTP:dTTP ratio that ultimately prevents the misincorporation of dUTP by RT [42,43]. Inactivation of the dUTPase in CAEV and feline immunodeficiency virus (FIV) leads to an increase in the mutation rate with the accumulation of guanine to adenine mutations. Both, dUTPase and *vpr-like* deletions appear to be implicated in the RT fidelity [44]. The dUTPase defective recombinant viruses have a less efficient replication in macrophages and fibroblast-like cells. This may confer an advantage to the host *in vivo* leading to a decreased viral replication and pathogenesis, since dUTPase is apparently required to eventually develop lesions such as those involved in bilateral carpal arthritis [42,44]. The effect of dUTPase defect has been recently proposed also in infections with a field isolate (genotype E1)—whose genome naturally lacks the dUTPase encoding region and the *vpr-like* gene—which do not appear to ever reach clinical stages, including carpal arthritis [45]. Another genotype E variant (E2), also lacking dUTPase, however, showed certain pathogenic features *in vitro* and *in vivo*. Strikingly, neither E1 nor E2 genome exhibit high mutation rates [46]. Overall, these findings, together with recent reports describing alternative dUTPase functions may re-issue the study on the role of dUTPase in lentiviral replication [47]. Additional mechanisms altering dUTP:dTTP ratio in the cell, the docking of cellular DNA glycosylates, or the counteraction of host apolipoprotein (APOBEC3) by viral *vif* [48] may also result in a major source of viral heterogeneity and define the infection outcome [39]. 

### 2.2. Recombination

Mutation alone is unlikely to explain the adaptive flexibility of lentiviruses. Recombination may occur frequently in the viral genome, as shown in the *env* gene of VMV strain 1514 *in vitro* and *in vivo* [49,50]. This mechanism of genetic diversification can efficiently shuffle mutations within a quasi-species; can rapidly assemble beneficial genetic combinations that would be difficult to generate by mutation alone; and can also effectively remove deleterious mutations. In contrast with the slow and steady changes caused by mutation, recombination is a much more powerful evolutionary force. First, recombination facilitates the repair of viral genomes. This can be due to physical repair at genome or breaks accumulating deleterious mutations via a copy choice mechanism [36]. In the absence of recombination, organisms tend to accumulate deleterious mutations that reduce viral fitness in each replication cycle. Multiple recombination events take place, with estimates generally falling between three and nine recombination events per genome per replication cycle [51].

Efficient recombination in retrovirus arises as a result of the co-infection of two viruses in the same cell and co-packaging of two copies of the RNA genome into each virion [52]. When a cell becomes co-infected by two or more different viruses, the corresponding RNA genomes can become co-packaged into the viral progeny. During subsequent reverse transcription, the viral reverse transcriptase readily switches between these two templates [53,54], which leads to the production of recombinant cDNA. If these templates are identical, then template switching will be genetically neutral and recombination will not be detected. Conversely, if these two genomes are non-identical, template switching will lead to viral recombination and progeny virus will be genetically distinct from the parental strains [36]. In natural infections, SRLV recombinations between different genetic groups (CAEV (B)-VMV (A)) [55] and between genetic variants of the same group (B1 CAEV) in goats have been identified [25]. Recombinant viruses may contain recombinant *env* genes involved in SRLV tropism, so that they may exhibit a modified range of targets (cell, tissue, and host species).

## 3. Phylogeny

The existence of high genetic variability among SRLV has given rise to numerous studies on the phylogenetic relationships among sequences obtained in different countries [56]. The classification of viral genotypes into groups and subtypes proposed in the last decade [27] in studies involving two long segments of the SRLV genome (*gag-pol* segment 1.8 kb; and *pol* segment 1.2 kb) is widely accepted at present. Accordingly, SRLV are classified into five groups (A–E). Following HIV classification criteria [57], genotype groups differ by 25% to 37% in their nucleotide sequences. However, genotypes A, B and E may further be distributed into different subtypes, differing in 15% to 27% of their sequence. Genotype D has been only described in Swiss and Spanish sheep, and only regarding *pol* sequences. However, there are no other studies confirming the existence of this genotype. Rather, phylogenetic analysis on additional (*gag*) sequences of the same (group D) isolates, classify these sequences with genotype A, suggesting that genotype D is in fact genotype A, exhibiting divergence in *pol* gene.

**Figure 1:**
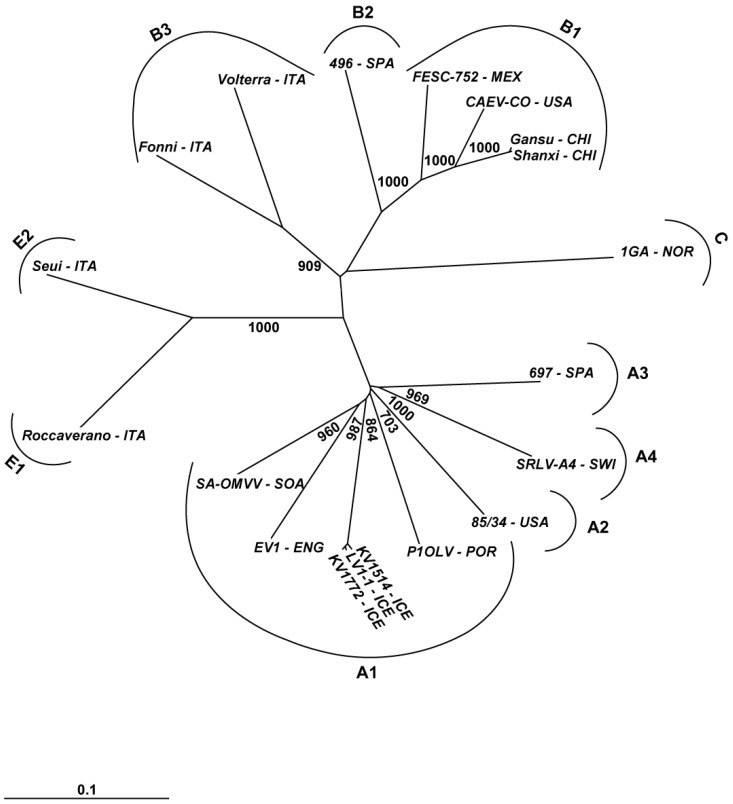
Phylogenetic tree involving SRLV complete sequences obtained from GenBank (accession numbers in bold and underlined) that include the name of isolate and country origin. Country abbreviations: CHI - China (Gansu **AY900630** and Shanxi **GU120138**); ENG - England (EV1 **S51392**); ICE - Iceland (KV1514 **M60610**, LV1 **M10608** and KV1772 **L06906**); ITA - Italy (Roccaverano **EU293537**, Seui **GQ381130**, Fonni **JF502416** and Volterra **JF502417**); MEX - Mexico (FESC-752 **HM210570**); NOR - Norway (1GA **AF322109**); POR - Portugal (P1OLV **AF479638**); SOA - South Africa (SAOMVV **M34193**); SPA - Spain (496 **FJ195346** and 697 **HQ848062**); SWI - Switzerland (A4 **AY445885**); USA - United States of America (CAEVCo **M33677** and 85/34 **AY101611**, **U64439**). The SRLV genotypes and subtypes are indicated.

Group A has so far 15 recognized subtypes, A1–A15, group B has three subtypes, B1–B3 and group E has only two subtypes, E1 and E2 (Table 1). Although SRLV infection in small ruminants is widely distributed in all continents [27,58], little information is available on the genetic variants circulating in different geographic regions (Table 1). SRLV complete genomes have been sequenced and are available in the GenBank derived from goat (CAEV-CO [59,60], 1GA [61,62], Gansu [63], Shanxi [64], FESC-752 [25], Seui [46] Roccaverano [45] and A4 [27] viruses) and sheep (Fonni [30], Volterra [30], 496 [65], SA-OMVV [66], KV1514 [67], KV1772 [68], LV1 [69], EV1 [70], P1OLV [71], 85/34 [72] and 697 [73] viruses) (Figure 1). In addition, partial sequences have been published in Brazil [74,75,76], Canada [77], Finland [78], France [27,79,80,81], Greece [82], Ireland [83], Japan [84], Netherlands [85], Poland [86,87], Russia [88], Slovenia [89], South Korea [90] and Turkey [29,30]. 

**Table 1:** Distribution of SRLV genotypes and subtypes which infect goats and sheep from different countries.

Country	Genotype A															Genotype B			Genotype C	Genotype D	Genotype E		References
	A1	A2	A3	A4	A5	A6	A7	A8	A9	A10	A11	A12	A13	A14	A15	B1	B2	B3	C	D	E1	E2	
Brazil	G/S															G							[74,75,76]
Canada		S														G							[77]
China																G							[63,64]
England	S																						[70]
Finland	S	S																					[78]
France	S					G/S										G/S	G/S						[27,79,80,81,91,92]
Greece					S																		[82]
Iceland	S																						[67,68,69]
Ireland	G																						[83]
Italy	G							G	G/S	G	G/S					G/S	S	G/S			G	G	[30,45,46,89,93,94]
Japan																G							[84]
Mexico																G/S							[25]
Netherlands	S																						[85]
Norway	S																		G/S				[61,62]
Poland	G/S											G/S	G/S			G	S						[86,87]
Portugal	S																						[71]
Russia																G							[88]
Slovenia					S									G	S	G							[89]
South Africa	S																						[66]
South Korea																G							[90]
Spain	S		S														S			G/S			[65,73]
Switzerland	S		G/S	G/S	G	G/S	G									G	S			S			[27,95]
Turkey		S	S		S				S		S							S					[29,30]
USA		S														G/S							[59,60,72,96]

G = Goat; S = Sheep.

According to the most recent phylogenetic information, mostly based on the gag gene, the types B, C and D and only nine of the 15 group subtypes A (A1, A3, A4, A5, A6, A9, A11, A12 and A13) infect both sheep and goats. Other groups and/or subtypes have only been described in one of the ruminant species: sheep (A2 and A15), or goats (A7, A8, A10 [93], A14, E1 and E2). However, as more information is generated, SRLV species restrictions are likely to dwindle (Table 1).

## 4. Tropism

SRLV tropism is linked to both, host genetics and viral genome heterogeneity, and can be studied according to differences in the targets addressed regarding: (a) host species (goats or sheep, or both); (b) tissue, differing according to the form of disease (mastitis, arthritis, encephalitis and/or pneumonia); and (c) cell type.

### 4.1. Host Species Restriction

Studies from several countries have confirmed that CAEV and VMV, originally established as specific pathogens in goats and sheep respectively, often cross the species barrier infecting the new host, persisting in it and spreading across the new host population. The first evidence of this transgression was obtained by experimentally infecting sheep and goats with CAEV or VMV [97,98,99,100,101,102]. Subsequently, evidence has been obtained from phylogenetic analysis of sequences derived from French sheep infected by viruses closer to CAEV (group B2) than to VMV (group A) [79,80,81,91,92]. On the other hand, molecular epidemiology studies have demonstrated direct virus passage from sheep infected with VMV-like strains (A4) to goats [95]. In line with this, the genotype B1 had been considered strictly caprine until the description of B1 infected sheep [103]. Thus, even if some genotypes might have been originally assigned to a single host species, the host species spectrum may be wider in nature.

Cell receptor usage explains, at least in part, different patterns in SRLV host restriction. As indicated above, the high variability of the ENV protein may be due to its interaction with the immune system and the generation of viral escape mutants [49,104]. Thus, changes in *env* may affect the ability of the virus to bind the putative cell receptor(s) and co-receptor(s). Differences in receptor usage have been observed between VMV and CAEV strains [105]. Also, differences in permissivity have been found *in vitro* between cell lines of heterologous origin (chicken, hamster, human, monkey and quail), which are permissive to SRLV infection and Chinese ovary hamster cells non-permissive to the infection [106,107]. Specific amino acid residues of the viral SU protein (see below) have been linked to cell receptor recognition [108]. Some of these residues may also be related to the presence of arthritis [104].

Another explanation for the host-specific viral restriction lies in the individual genetic background. Besides adaptive immunological correlates of protection according to immunization studies [109], cell molecules of the innate immunity may be involved in delimiting host restriction. Among these, APOBEC and TRIM5 are the most widely studied, as they act in different species directly by mutating viral genome and interacting with viral capsids, respectively, showing species specificity [110,111]. However, the virus has developed mechanism to counteract proteins of different host species. VMV studies have shown that the viral Vif protein can neutralize a broad number of A3Z3 proteins (APOBEC) irrespective of the species of origin (sheep, humans, macaques, cows and cats) [112]. 

### 4.2. Viral Genetics by Organ and Tissue

Amino acid motifs conserved at specific viral protein positions involved in the viral entry (“signature patterns”) [113] have been found in individuals presenting the same form of disease in HIV [114] and FIV [115] infections. Signature patterns that define organ tropism (disease form) have not been found so far in SRLV infections [116,117]. At the single individual level infected with a lentiviral quasi-species, each particular virus variant may be confined to one particular compartment, organ or tissue within the individual. In HIV infections it is known that these viral populations change to adapt to local media within the individual through genetic drift (founder principle) or selection pressure. However, when the flow of genes between viral subpopulations within the individual is significantly restricted, then each subpopulation can become genetically distinct. This phenomenon named compartmentalization [115,118,119] may be derived from micro-evolution and/or the presence of various but phylogenetically related genotypes [120,121,122]. High viral mutation rate *in vivo* can quickly enhance the genetic distance between subpopulations. Also, differences in selective pressures imposed by the immune system can result in divergent evolution of the virus, affecting cell tropism, phenotypic characteristics and/or pathogenesis, as shown in HIV infections [123,124,125,126]. 

Regarding the particular viral genetic region involved in tropism, LTR and *env* have been the most widely studied in lentiviral infections. LTR, being a non-coding region responsible for docking important cellular transcription factors, is essential for viral replication and may affect viral phenotype in cell culture [127,128]. This LTR function is achieved through LTR transcription factor binding sites within the U3 region, some of which are related to cell tropism [129]. Tissue tropism has been evaluated *ex vivo* by studying SRLV LTR sequences in different tissue samples but, again, no signature patterns have been found so far [130]. Alternatively, in other lentiviruses, genomic regions such as hypervariable regions of the *env* gene involved in compartmentalization have been found implicated in tropism. This is the case of the V3 hypervariable region of *env* in HIV infections, being determinant of cell tropism and replication efficiency as it relates to the fusion and virus adsorption to the cell in macrophage-tropic strains [113]. In SRLV genomes, five variable (V1 to V5) and four conserved (C1 to C4) regions in the envelope protein have been identified [108,131]. The SRLV *env* hypervariable region (V4) is structurally and functionally analogous to the V3 region of HIV [108,132,133]. To colonize different organs (lung, mammary gland, brain, joints) SRLV can undergo variations in the V4 region of the *env* gene during early infection giving rise to different viral subpopulations, as shown in other lentiviruses [132]. A study on SRLV compartmentalization in the mammary gland of goats and sheep showed the presence of different viral sequences in the V4 region of the *env* gene compared to blood-derived cells and colostrum. It was proposed that the mammary gland was colonized with a “founder virus” which possibly represented the most common variant circulating (dominant) in the blood at the time of infection. Alternatively, it was proposed that the initial mechanism could be totally different to compartmentalization and would reflect a selection pressure caused by a particular cellular tropism or related to the immune system action exerted against the virus in this compartment [116].

In another study, SRLV genotype A sequences from sheep suffering from neurological disease (visna) showed a clear tissue compartmentalization in the central nervous system (CNS) and other organs such as lung and mammary gland, related to horizontal and vertical transmission of SRLV infection, respectively. Bayesian approach inferences have suggested that proviruses from alveolar macrophages and peripheral blood mononuclear cells (PBMC) represent the most probable common ancestors (infecting viruses) in the animal. Likely, PBMC become infected after the intake of virus or infected cells/particles through respiratory and/or mammary secretions, including colostrum/milk. Neuroinvasion in the visna outbreak involved microevolution after initial infection with SRLV [117]. Of note, the findings on SRLV diversification, adaptation and/or compartmentalization in the brain may be independent of those observed in lymphoid tissues or other organs, where the virus is exposed to more abundant immune pressures affecting viral replication [134].

### 4.3 Cellular Tropism

The members of the lentivirus genus differ in cellular tropism and disease development, being distributed into two groups. One group includes HIV, FIV and simian immunodeficiency virus (SIV), all of which replicate in macrophages and lymphocytes causing acquired immunodeficiency syndrome and specific-organ disease affecting lungs, CNS and gastro-intestinal tract. The other group includes equine infectious anemia virus (EIAV) and SRLV, both of which replicate primarily in macrophages (lymphocytes are not infected) and cause a disease affecting specific organs, mainly lung, mammary gland, CNS and/or joints [127,135].

In the VMV epidemic in Iceland, the most common clinical signs corresponded to the maedi (respiratory form) and the visna (nervous form) was only reported in herds with high prevalence of maedi for several years. This was originally attributed to the cell tropism of the virus rather than the genetic variation of sheep, because there was only one sheep breed in Iceland [136,137]. Subsequently, it was shown that the cellular tropism of VMV strains from sheep with visna appeared to be different from those isolated from sheep with maedi. The virus isolated from brain cells replicated more rapidly in sheep choroid plexus compared to isolates from lung and this difference was related to differences in *env* and LTR regions [138]. 

*In vivo* SRLV have a marked tropism for cells of the monocyte/macrophage lineage and dendritic cells [139,140], the virus infects monocytes, so these cells are permissive for viral entry and genome integration (provirus), but the infection remains latent until the cell differentiates into macrophage [139,141,142]. This maturation enhances the expression of the transcription factors c-Fos and c-Jun, which bind to the AP-1 and AP-4 promoter binding sites of LTR triggering transcription, and consequently viral replication and productive infection [143]. Thus, SRLV replicate in mature macrophages or related tissues rather than in circulating monocytes, the latter acting as a “Trojan horse” [144] 

SRLV are highly variable genetically and antigenically and also show phenotypic differences *in vitro.* Specifically, isolates may differ from each other in the ability to selectively and productively infect particular cell types and also in the capacity to induce a cytopathic effect. Accordingly, SRLV may be classified phenotypically as rapid/high or slow/low. The rapid/high strains replicate rapidly, inducing the formation of syncytia, cell lysis, and reaching high titers. In contrast, the slow/low viruses grow slowly and at a low titer. Frequently, sheep isolates belong to the first type; whereas isolates from goats show a slow/low phenotype. However, SRLV strains that show an intermediate phenotype between the most extreme phenotypes of VMV-like and CAEV-like viruses have been isolated from both sheep and goats [145,146].

Because SRLV show *in vitro* high viral production in permissive cells such as macrophages, these cells are used routinely for isolating the virus, even though they are terminally differentiated [147]. Fibro-epithelial synovial membrane cells and choroid plexus cells derived from goats or sheep are also routinely used for *in vitro* production of SRLV [96,148]. Other cell types permissive to SRLV infection are from lung explants [149], skin fibroblasts [140], spleen and corneal cells [150,151], testicle cells [152], goat endothelium [153,154], mammary gland epithelial cells (TIGMEC cell line) [155,156], granulose cells [157], oviduct cells [158], microglial cells [159,160], tubular epithelial cells [161], hepatocytes [162], cardiac myocytes [162] and the third eyelid cells [163]. However, the restricted viral antigen expression and the low number of infected cells found in some of these cells should be taken into account before interpreting the biological relevance of these cells in SRLV persistence [164]. Experiments employing pseudotyped vectors (vaccinia virus or murine leukemia virus-based) with SRLV ENV indicate that ENV-based interaction with cells renders them permissive to virus entry. This is the case of cell lines Hela, BHK-21 and others from different origins (human or other species) [106,107], suggesting that these cells have a receptor for ENV and that the virus enters the host cell by fusion of the membrane receptor to viral ENV SU protein gp135 [165]. However, this permissiveness at the entry step [166] may not be associated with a capability of productive infection or to a possible natural susceptibility. Although in previous SRLV studies restriction had been attributed to the lack of functional receptors [167], post-entry restriction factors such as those interacting with Gag [110] rather than Env proteins may have been responsible for the lack of productive infection.

Different attempts have been made to identify and characterize SRLV receptor(s). Candidate SRLV receptor molecules include: a membrane-associated proteoglycan substituted with one 30 kDa chain (s) chondroitin sulfate glycosaminoglycan [168]; molecules of the major histocompatibility complex class II (MHC class II), as preincubation with MHC class II antigens inhibits VMV infection (even though antibodies specific to antigens of MHC class II do not inhibit infection) [169]; CD4 and CXCR4 molecules, which have been proposed as optional auxiliary components for VMV receptor (or receptor complex) that facilitates membrane fusion events mediated by VMV [166,170]; and a complex comprising three membrane proteins of 15, 30 and 45 kDa, identified as VMV binding proteins [171]. However, none of these molecules has been established as the main/essential receptor. Analysis of somatic hybrid cell lines (CHO cell panel containing different mouse chromosomes) permissive to VMV entry has shown that the gene for cellular receptor is in the murine chromosome 2 or 4 (although involvement of chromosomes 6 and X is not excluded). On the other hand, it also showed using hybrid cell lines hamster × sheep and hamster × human [172] that the VMV receptor gene maps to sheep chromosome 3p (OAR-3p) and a region of human chromosome 2 (HAS-2p25 > q13) has retained synteny with sheep chromosome 3p. These regions do not include any gene of known receptor or co-receptor of lentiviruses, which would indicate that SRLV receptor uses a different molecule to infect human cells. Apparently, SRLV may use different cell receptors likely depending the cell it encounters [105]. Recently, the mannose receptor (MR, codified in mice by chromosome 2) has been described in sheep and identified as a potential SRLV receptor. Accordingly, three cell phenotypes have been proposed in small ruminants regarding the expression and usage of MR as SRLV receptor: cells using this molecule as the main receptor (synovial membrane cells), cells having this and at least another main SRLV receptor(s) (macrophages) and cells lacking MR and using other SRLV receptor(s) instead (skin fibroblasts) [173]. Interestingly, the presence of this receptor has been associated with the evolution of disease, as its expression increases in target organs exhibiting the most severe lesions in SRLV clinical infections [174]. 

## 5. *In Vitro* Diagnosis

Different laboratories have applied direct diagnostic methods such as viral isolation, a very laborious method whose performance can be hampered by the lack of permissive cell lines and limited viral production in cultured cells [96]. Isolation of virus attempted from leukocytes of infected animals often fail, indicating that a negative result is not always reliable [175]. Although this method is not suitable for large-scale studies, it can be performed by co-cultivation (commonly of blood monocyte derived macrophages with fibro-epithelial cells), whereby the detection of cytopathic effect or reverse transcriptase activity indicate the presence of virus [65]. Similarly, immunohistochemical methods, allowing the detection of viral protein antigens by specific antibodies in histological samples, smears or cytospin preparations, are useful in research and confirmatory studies, but are not commonly applied in differential routine diagnosis on live animals, because of the cost, low availability of reagents and samples and limited sensitivity of some of these techniques [175]. Also, *in situ* hybridization methods have been employed for histologic studies, but this laborious procedure is only used for research purposes [176,177]. Although PCR-based diagnosis is the most common of the molecular techniques applied in SRLV diagnosis *in vitro* (see below), indirect methods such as serological diagnosis to detect antibodies to SRLV are overall the most commonly applied approach to detect infection. However, there are problems inherent to serological diagnosis. These include the high genetic/antigenic variability existing in laboratory and field strains, the presence of maternal antibodies, the relatively long lag period between infection and antibody production (serological gap) and the intermittent seroconversion (by fluctuating titers throughout the animal’s life). Antibody production has been detected by techniques such as agar gel immunodiffusion (AGID), the enzyme-linked immunosorbent assay (ELISA), radioimmunoprecipitation (RIPA) and Western blot (WB) [178,179,180]. Both RIPA and WB are mainly used for confirmation [181]. AGID is highly specific but relatively insensitive [178] and often linked to subjective interpretation, inapplicability for the determination of antibodies in milk and lack of automation, motivating its replacement by ELISA methods of relatively low cost and easy implementation and interpretation [2,179,181]. 

### 5.1. ELISA Tests

In spite of the antigenic variability, cross-reacting antibodies between VMV-like and CAEV-like antigens have been described [182] and have enabled for years the control of SRLV-induced disease. Sheep and goats remain infected for life, but many seropositive animals may never show clinical signs of SRLV infection. Antibodies do not protect against the disease and are mainly indicators of infection. The virus escapes from the immune attack even in the presence of neutralizing antibodies, as proviral DNA integrates into host cell genome and viral mutations take place during viral replication [183]. On the other hand, a seronegative animal cannot be strictly considered free of infection, either because antibody titers are below detection level before seroconversion, or because fluctuation in antibody levels occurs throughout the animal’s life. Newborns infected at birth have maternal antibodies (through colostrum/milk intake) for at least two or three months. Thereafter, they are usually seronegative until they seroconvert between six and twelve months of age. Performing diagnostic tests immediately before pregnancy does not necessarily ensure that mothers will not seroconvert after parturition. Thus, infected seronegative animals constitute a potential source of infection through vertical and horizontal transmission [179,181].

To date, no single technique or test can be proposed as “gold standard” to determine the infection status of the animal [175]. Competitive ELISA methods (cELISA CAEV of VMRD Inc. Pullman, WA) [184,185] using monoclonal antibodies to viral envelope protein (ENV-SU, gp135) epitopes have been developed [186] and indirect ELISAs have been frequently applied [187,188], although few of them have been compared internationally for both sheep and goats [189,190,191]. Currently available indirect standard ELISAs are used in different formats and designs. Examples are those using either whole virus antigens such as the AG-CHEKIT (CAEV/MVV kit, IDEXX Switzerland AG, Liebefeld, Bern, Switzerland) [192] or recombinant proteins plus a peptide, specifically, the GAG p25 recombinant protein and a TM peptide derived from genotype A, as used in assays Elitest-MVV (HYPHEN Biomed, Neuville-sur-Oise, France) [193] and Pourquier (ELISA Maedi-Visna/CAEV serum verification Institut Pourquier, Montpellier, France). But in some cases, monostrain assays may not cover the whole SRLV antigenic spectrum and fail to detect genotype E [188], B2 [194] and A4 infections [195]. Besides the viral p25 protein included in the test, p14 and p17 proteins also present immunodominant epitopes and could be used for differential VMV/CAEV diagnosis [196,197]. Gag proteins (p25) have been used in early diagnosis and detection of cross-reacting antibodies for more than 20 years [179]. However, new genotypes have emerged [30,45], broadening diagnostic possibilities, leading to the development of new standard indirect ELISA tests, such as the one based on a mixture of *gag* and *env* peptides of three different SRLV genotypes (A, B and E) (IN3 diagnostic. Eradikit® SRLV indirect ELISA for Small Ruminant Lentivirus).

The ENV TM protein has an added value in diagnosis of long-term infections such as those present in diseased animals [198] and therefore is included in most subunit assays. SU peptides have also been widely used in serological diagnosis and, due to high variability of SU proteins, these peptides are being useful to serotype and perform strain-specific diagnosis [198]. Synthetic peptide ELISAs [199] have been evaluated by our group, outlining their utility in field sample diagnosis of animals from neurological [73] and arthritic [65] outbreaks.

Antibody detection technology is certainly a valuable tool for determining the infection status of a herd/flock, although in some areas with limited resources this technology may be somewhat expensive if performed at the individual level. Therefore, strategies for detecting antibodies and infection in the flock, involving the use of bulk milk [200,201,202] or pooled serum [203] and semen samples [191,204] are particularly useful in these areas.

### 5.2. Polymerase Chain Reaction (PCR)

Molecular techniques of use in SRLV diagnosis include the heteroduplex mobility assay (HMA) [80], which may help to a genotypic characterization of circulating strains within the flock, a loop-mediated isothermal amplification technique [64] and polymerase chain reaction (PCR) procedures, the most commonly used to directly assess the presence of viral nucleic acid. Conventional PCR for diagnostic purposes has been more extensively studied than the real-time PCR (rtPCR). The introduction of rtPCR represents a breakthrough in molecular diagnosis [205,206]. The PCR and the early detection of amplicons have been combined in this technique [206,207]. The rtPCR has been useful for detection and quantification of viral nucleic acids in different cells or tissues, although its use in SRLV routinary diagnosis is not widespread [207,208,209]. Primers designed in almost all SRLV genetic regions [205] have yielded reliable and fast results. The rtPCR diagnostic approach presents a decreased risk of cross-contamination and has a high sensitivity using SYBR Green I or TaqMan-based methods [207]. Furthermore, the use of oligonucleotide probes helps to increase rtPCR specificity [205]. However, this technique may work only on a restricted number of strains and its use in field diagnosis may present difficulties due to variation in the primer binding site. 

The first conventional PCR protocols designed to detect SRLV were published in the early 1990s [210,211]. PCR was considered initially a successful tool in SRLV diagnosis, due to its ability to directly detect viral nucleic acids, either in the infected cell as a provirus (DNA) or in exudates that contain free virus particles (RNA; reverse transcription-PCR) [212]. In addition to conventional PCR, variants of this technique have appeared involving two (or more) amplification rounds (nested-PCR and seminested-PCR) in order to increase the sensitivity [213,214,215,216,217]. Different studies have shown the utility of conventional PCR to detect SRLV infections in different animal samples, namely PBMC [214,218,219,220], peripheral blood leukocytes (PBL) [213,221], milk or mammary secretions [212,214,218,219,220,222], semen [223,224], synovial fluid [218] and other tissues [214,222,225]. The efficiency of PCR depends mainly on the specificity of the primers designed, the choice of the amplified target viral region, and the sensitivity of the technique [35,127,226]. The low viral load existing in infected animals, the low proportion of permissive cells in some tissues (there is one infected monocyte in 10^4^–10^5^ PBMC) [206] and the high genetic heterogeneity may hamper PCR diagnosis of SRLV [2,226]. Furthermore, the cell type may affect PCR performance. However, PCR has the advantage over serological methods of detecting infection in animals with colostral antibodies. In milk samples from sheep and goats (small ruminants) a seminested-PCR method based on the *pol* region (pol-seminested-PCR) has yielded positive results in all the infected small ruminants tested (the seropositives determined previously by a serological test and a seronegative animal) [212]. Similar PCR sensitivities have also been found in PBMC, milk cells and synovial fluid [218]. Here, however, are also studies reporting a decreased sensitivity when using milk samples (23.6%) relative to blood samples (33.3%), using a *gag*-PCR [219] or a LTR-PCR procedure [222]. Target sequences for PCR primers design are widespread throughout the SRLV genome including LTR, *gag*, *pol* and *env* regions [164,212,222,224] and lead to different sensitivity and specificity values. In PCRs amplifying segments of the SRLV *gag* and *pol* regions, the *gag*-PCR was more sensitive than *pol*-PCR [211]. However, the LTR-PCR may be more sensitive than PCRs based on *gag* and *pol* regions [213,222,226]. In a study that examined four pairs of primers designed in LTR, *gag* (capsid and matrix) and *env* regions using DNA from cells infected with virus from various geographical areas, PCR primers targeting the LTR region, *gag*(capsid), *env* and *gag* (matrix) amplified 100%, 62.5%, 25% and 12.5% of the samples, respectively [227]. This is in contrast with another study showing that a PCR based on *env* sequences could be more efficient at detecting infected animals (93.3%) compared to *pol*-PCR which only detected 60% of animals infected. Despite its presumed high variability, the *env* region can be suitable for diagnosis by PCR (Table 2) [224].

In SRLV infections, PCR-positive reactions are often found amongst seronegative animals (false negatives) (Table 2) [219,228], which later seroconvert, thereby demonstrating a lack of sensitivity of the serological methods compared to PCR under particular conditions [195]. 

**Table 2:** Different PCRs techniques, target DNA samples, regions of the viral genome used for PCR design and sensitivity of the PCR method for detection of infection by SRLV.

PCR type	DNA or cDNA source	Primers location into the viral genome	Sensitivity test	References
cPCR	Cell culture	*gag* and *pol*	*pol*-PCR was more sensitive than *gag*-PCR	[210]
snPCR	PBL	*pol* and LTR	LTR-PCR was more sensitive than *pol*-PCR	[212]
nPCR	PBMC	*gag* and *pol*	*gag*-PCR was more sensitive than *pol*-PCR	[213]
snPCR	PBMC	*gag*	*gag*-PCR was less sensitive than AGID	[214]
snPCR	Cell culture and PBMC	*pol*	High sensitivity, by using degenerate primers	[215]
nPCR	PBMC	*gag*	*gag*-PCR was more sensitive than AGID in seronegative animals	[216]
nPCR	PBL and blood	*gag* and LTR	*gag*-PCR was more sensitive than LTR-PCR	[220]
cPCR	Semen	*env* and *pol*	*env*-PCR was more sensitive than *gag*-PCR	[222]
cPCR	PBMC, milk cells and tissues	LTR	LTR-PCR had a sensitivity of 98% with regard to AGID and ELISA	[223]
cPCR	Cell culture	LTR, *gag* and *env*	LTR-PCR was more sensitive than *gag*-PCR and *env*-PCR	[226]
cPCR	PBMC	*gag*	*gag*-PCR was less sensitive than AGID	[228]
cPCR	Cell culture and PBMC	*gag*	*gag*-PCR was more sensitive than the ELISA and WB	[229]
nPCR	PBL	*gag*	*gag*-PCR increases its sensitivity when used along with hybridization	[230]

cPCR: conventional PCR; snPCR: seminested PCR; nPCR: nested PCR; PBMC: peripheral blood mononuclear cells; PBL: peripheral blood leukocytes; AGID: agar gel immunodiffusion; ELISA: enzyme-linked immunoassay; WB: western blot.

Studies based on *gag*-PCR diagnosis reveal that this method may detect infection in 88.3% of sheep and goats that are seropositive by AGID and in 11% of seronegatives (67% of animals eventually seroconverted) [229]. In line with this, a *gag*-seminested-PCR procedure allows the detection of 70% of the AGID positive sheep and 11% of seronegative animals [215]. Comparative studies on the sensitivity of the PCR with regard to serological tests (ELISA and AGID) have shown that LTR-PCR is less sensitive than the ELISA and AGID. However, PCR showed a specificity of 100% (like AGID), whereas ELISA was less specific (59%) [230]. LTR-PCR specificity is maintained (100%) in different tissues, but sensitivity values change according to the tissue under study [83.5% (peripheral blood leukocytes), 66.7% (milk cells) and 88% (tissues)], being less sensitive than the ELISA and AGID [222]. 

Since PCR assays may fail to detect virus when virus load is below the assay threshold, PCR sensitivity may increase upon co-cultivation of PBMC from infected animals with permissive fibroblasts *in vitro* [225,231,232] or by using probes in the Southern blot [214,219] or *in situ* hybridization [228,233] techniques. The use of strain-specific and sequence-degenerated primers could also improve sensitivity, as shown by Elthair and collaborators [216] using a *pol*-seminested-PCR on AGID-seropositive animals infected with two viral subtypes (genotypes A and B). The percentage of *pol*-PCR positives in this study was 62.2% and 30.5% for types A and B, respectively. In addition, the *pol*-PCR detected 38.2% of positive animals among AGID seronegatives [216].

Although different studies indicate that in general the PCR methods tend to be less sensitive than serological tests (particularly ELISA tests) (Table 2) [213,220], PCR testing can detect infected animals prior to seroconversion [213,219,228,234,235]. According to SRLV pathogenesis, provirus should be present prior to the antibody response to the virus during initial infection of adult animals, but this is not always detected. In studies comparing PCR with serological test (AGID and ELISA) diagnosis, agreement (positive concordance) ranged from 70% to 94.7%, whereas disagreement (negative concordance) ranged from 87.5% to 100% [181,218,219,222,224,229]. This suggests that a combination of serology and PCR might be optimal for detecting the infectious status of a population [217,230,236]. Furthermore, PCR products can be systematically sequenced [27,94], which may allow a better design of primers for new PCRs with a wide range of recognition. The development of a single PCR to detect all SRLV may still be utopian, although attempts to cover a large spectrum of genomes by including different sets of primers in the assay have been made [27]. 

## 6. Concluding Remarks

The basic mechanisms of mutation and recombination are complex biological processes, but they may help to understand the true role of viral diversity in SRLV pathogenesis. Virus and host genetic and microenvironmental factors involved in cell, tissue/organ and host tropism of these viruses are pivotal issues in current investigations. Genetic and antigenic variations of the virus represent challenges in SRLV diagnosis. The success in avoiding SRLV infection spread depends largely on early detection and culling of infected animals in the heard/flock. Detection of specific antibodies in serum or milk by ELISA, typing the circulating strains by local strain-based ELISAs, HMA or PCR-sequencing techniques, and use of PCR for confirmatory purposes are solid technological approaches of practical use for reducing the risk of misdiagnosis in different areas. Finally, diagnostic methods suitable to both sheep and goats should be implemented in joint programs for these species for successful SRLV control and eradication strategies.
